# Somalia's new community health strategy: Strengthening primary health care and health system resilience

**DOI:** 10.1016/j.pmedr.2026.103596

**Published:** 2026-08-07

**Authors:** Maryama Ahmed Ali, Abdirahman Mohamed Adan, Ahmed Mohamed Omar, Ahmed Osman Ibrahim, Yakub Burhan Abdullahi, Mohamed Sharif Abdi

**Affiliations:** a Faculty of Health Sciences and Tropical Medicine, Somali National University, Mogadishu, Somalia; b Center for Health Research and Innovation, Somali National University, Mogadishu, Somalia; c Benaadir Research, Consultancy and Evaluation Center, Somalia; d Faculty of Health Science, Salaam University, Mogadishu, Somalia

## Abstract

**Background:**

Somalia has introduced a new Community Health Strategy to strengthen primary health care (PHC) and improve health system resilience. The strategy addresses persistent gaps in service delivery, limited access to essential health services in rural and underserved communities, and inadequate capacity to respond to public health emergencies.

**Methods:**

This correspondence presents a descriptive policy analysis of Somalia's newly launched Community Health Strategy using national policy documents and relevant peer-reviewed literature to assess its priorities and implications for community-based health service delivery.

**Results:**

The strategy prioritizes expansion of community health worker programs, integration of preventive and promotive services, strengthened referral pathways between communities and primary health facilities, improved workforce capacity, enhanced supervision, and stronger coordination among government and implementing partners. It also emphasizes preparedness for disease outbreaks and other public health emergencies to improve health system resilience.

**Conclusions:**

Somalia's Community Health Strategy represents an important step toward strengthening primary health care and building a more resilient health system. Effective implementation could improve equitable access to essential services, enhance community-level disease prevention and response, and accelerate progress toward universal health coverage.

Community health systems are a lifeline in fragile and conflict-affected settings, where insecurity, displacement, and poverty systematically exclude millions from essential care. Somalia exemplifies this reality: less than one-third of the population can reach a functional primary facility, its Universal Health Coverage (UHC) index is estimated at 25–33.5, and health worker density remains critically low at 0.11 clinicians per 1000 people (Dirie et al., 2025; Nikoloski et al., 2025). Against this backdrop, Somalia's new community health strategy, anchored in scaling up community health workers (CHWs), especially female CHWs, offers a pivotal opportunity to reorient the health system toward resilient, community-centred primary health care (PHC) (Dirie et al., 2025; Nikoloski et al., 2025).

The strategy aligns with the National Transformation Plan (2025–2029), which prioritizes PHC expansion, rural facility rehabilitation, and female CHW deployment to extend immunization, antenatal, and health-promotion services to remote settlements (Dirie et al., 2025). Evidence from the Female Community Health Workers program shows that 12-month–trained CHWs, each covering 600–1000 people and conducting 5–7 home visits daily, can deliver antenatal care, childhood immunization, treatment of common illnesses, and reproductive health services while tracking births, deaths, and population movements (Nikoloski et al., 2025). This model directly addresses stark service gaps: only 7% of pregnant women receive four or more antenatal visits, with nomadic and rural women facing 70–90% lower odds of adequate care than urban women (Abdi et al., 2025).

CHWs are also central to surveillance and outbreak preparedness. During the first year of COVID-19, community-based surveillance accounted for one-third of all suspected cases, with higher positivity than facility detection and far greater contact tracing yield (13,279 vs. 1937 contacts) (Nyagah et al., 2023). In a context of recurrent measles, cholera, and polio outbreaks, fueled by climate shocks, <0.4 doctors, nurses, and midwives per 10,000 population, routine immunization completeness around 20%, and ≥ 2.6 million internally displaced persons (IDPs), integrating CHWs into climate-informed surveillance and mobile vaccination is indispensable (Hussein et al., 2025).

High zero-dose prevalence (44% of children 12–23 months with no DPT1, exceeding 60% in some regions) underscores the need for targeted, community-based delivery to the poorest, rural, and IDP communities (Halane et al., 2025).

However, the promise of the new strategy is constrained by systemic challenges. Somalia's UHC score is the lowest among 120 countries assessed, reflecting chronic underfunding, fragmented infrastructure, and high unmet need around one-third of the population forgoes care due to cost (Nikoloski et al., 2025). More than 80% of health funding is externally sourced, with the Ministry of Health receiving ≤7% of the national budget; parallel donor systems fragment governance and threaten sustainability as recent cuts reduce essential food, health, and WASH services (Hussein et al., 2026). Insecurity, weak federal–state coordination, and structural barriers in IDP settings including distance, transport, facility closures, and lack of privacy further limit maternal and child health service use (Said and Kicha, 2024).

To translate the community health strategy into durable gains, several actions are critical. First, federal and state authorities should embed CHWs within PHC teams, with standardized training, supervision, remuneration, and reliable supply chains, prioritizing rural, nomadic, and IDP populations (Dirie et al., 2025; Nikoloski et al., 2025; Hussein et al., 2025). Second, community-based surveillance and immunization should be climate- and conflict-sensitive, linking CHWs to integrated outbreak operations centres and hydro-meteorological early warning (Nyagah et al., 2023; Hussein et al., 2025).

Third, domestic resource mobilization and pooled, on-budget donor financing must progressively increase the share of public funding for PHC, reducing dependence on fragmented vertical projects (Nikoloski et al., 2025; Hussein et al., 2026). Finally, governance reforms should strengthen federal–state accountability, data systems, and meaningful community participation, particularly of women leaders and IDP representatives (Dirie et al., 2025; Hussein et al., 2026; Said and Kicha, 2024).

Investing in Somalia's community-based health system is both a moral imperative and a strategic pathway to UHC and resilience. The new community health strategy offers a timely platform; it now requires sustained political commitment, predictable financing, and rigorous implementation to ensure that no community is left behind.

## CRediT authorship contribution statement

**Maryama Ahmed Ali:** Writing – review & editing, Writing – original draft. **Abdirahman Mohamed Adan:** Methodology. **Ahmed Mohamed Omar:** Validation. **Ahmed Osman Ibrahim:** Conceptualization. **Yakub Burhan Abdullahi:** Supervision. **Mohamed Sharif Abdi:** Visualization, Validation, Conceptualization.

## Clinical trial number

Not applicable

## Consent for publication

Not applicable.

## Ethical approval

This correspondence is based exclusively on publicly available policy documents and published literature. It did not involve human participants, identifiable data, or animal subjects; therefore, institutional ethical approval and informed consent were not required.

## Ethics declaration

The author(s) declare(s) that the study does not involve humans nor animal subjects.

## Funding

The authors received no financial support for the research, authorship, or publication of this article.

## Declaration of competing interest

The authors declare that they have no known competing financial interests or personal relationships that could have appeared to influence the work reported in this paper.

## Data Availability

No data was used for the research described in the article.
